# Diabetes disease prediction system using HNB classifier based on discretization method

**DOI:** 10.1515/jib-2021-0037

**Published:** 2023-02-23

**Authors:** Bassam Abdo Al-Hameli, AbdulRahman A. Alsewari, Shadi S. Basurra, Jagdev Bhogal, Mohammed A. H. Ali

**Affiliations:** Centre for Software Development & Integrated Computing, Faculty of Computing, Universiti Malaysia Pahang, Pahang 26600, Malaysia; Computing & Data Science Department, School of Computing and Digital Technology, Faculty of Computing, Engineering and the Built Environment, Birmingham City University (City Centre Campus), Curzon Street, B4 7XG, Birmingham, UK; Department of Mechanical Engineering, Faculty of Engineering, University of Malaya, 50603 Kuala Lumpur, Malaysia

**Keywords:** classification, data mining, discretization, HNB, Pima dataset

## Abstract

Diagnosing diabetes early is critical as it helps patients live with the disease in a healthy way – through healthy eating, taking appropriate medical doses, and making patients more vigilant in their movements/activities to avoid wounds that are difficult to heal for diabetic patients. Data mining techniques are typically used to detect diabetes with high confidence to avoid misdiagnoses with other chronic diseases whose symptoms are similar to diabetes. Hidden Naïve Bayes is one of the algorithms for classification, which works under a data-mining model based on the assumption of conditional independence of the traditional Naïve Bayes. The results from this research study, which was conducted on the Pima Indian Diabetes (PID) dataset collection, show that the prediction accuracy of the HNB classifier achieved 82%. As a result, the discretization method increases the performance and accuracy of the HNB classifier.

## 1 Introduction

Numerous kinds of research is being conducted on the application of data mining to healthcare datasets, and many of these applications have attempted to enhance the classification accuracy. Data mining is claimed to classify variables that affect the doctor’s ability to prescribe and contribute to new medications and a successful diagnosis [1].

Diabetes becomes the top deadly disease globally, and it contributes to deaths of stroke, heart attack, blindness, kidney failure, amputation, and liver disease worldwide. According to the World Health Organization (WHO) report, about 422 million adults worldwide have diabetes, with the majority being women; 5.2 million deaths are due to diabetes and high blood glucose. The report shows that one in eleven people get diabetes, and based on this report, the figure is expected to rise to 592 million by 2035. Machine learning is a modern technology that expands with a variety of implementations and can be one of the main components of intelligent information systems. It requires compact generalizations derived from vast repositories of datasets of historical information to be used as knowledge in various practical ways; for example, it is used in automated processes such as systems of experts or explicitly used to communicate with the experts of humans and for educational purposes [2]. Diabetes is one of the most severe challenges in the health sectors facing most developed and developing countries today. There are several datasets for diabetes, however, Pima Indian Diabetes Database (PIDD) at the UC – Irvine Machine Learning Laboratory is a publicly available database [3], which has become a standard for training and testing data mining algorithms to verify their accuracy in diabetic status prediction [4], [5], [6]. The Classification system will aid physicians to investigate disease conditions by working as a system or depending on medical consultations, whether these conditions are likely to get diabetic or not. Zhang and Jiang have suggested the Hidden Naïve Bayes (HNB) algorithm in 2009 [5]. The Hidden parent is generated in HNB for each attribute, which incorporates the properties of all other attributes [7, 8]. HNB indicates remarkable performance and predictive accuracy than other traditional algorithms of classification.

This study involves attempts to increase prediction accuracy and predict potential outcomes for diabetes disease based on the data mining techniques and knowledge obtained from recorded remarks by probabilistic approaches using the HNB classifier.

## 2 Related works

Classification algorithms (or classifiers) performance is calculated by their accuracy of classification (or error rate) in a manner that specific classifiers, such as Naïve Bayes and Decision Tree [9]; and HNB will equally produce an estimate of the class probability *P*(*c*|*i*), which is the likelihood of instance “*i*” in class *c*.

Robert Cattaral [10] and Iyer Aiswarya et al. [11] have conducted several comparisons using data mining techniques and different classification algorithms, including the HNB algorithm on the Pima dataset (PIDD). The results reviewed that the HNB compared with two algorithms (AODE, Selective Bayesian Classifiers SBC) applied to the PID dataset, which produces higher prediction accuracy. They concluded that the HNB outperforms Boosted Naive Bayes (BNB), Naive Bayes (NB), SBC, NB Tree, Tree augmented naive Bayes (TAN), and AODE in ranking; and Support Vector Machines (SVM), Decision Tree.

Jabbar et al. [12] conducted the Hidden Naïve Bayes and Naïve Bayes assessment on the discovery and prediction of the heart disease dataset on the Statlog data set. The proposed model assumes that the presence of one feature in a particular class is not related to the presence of any other feature to enhance the efficiency of HNB. Experimental results showed that HNB recorded the highest predictive accuracy 100% and outperformed Naïve Bayes; thus, this result was comparatively better than other approaches. An automatic heart disease prediction system with HNB model assistance is more reliable.

Furthermore, the Naïve Bayes and Decision Tree algorithms used the percentage split technique to study hidden patterns in the PID dataset, A comparison was made of the performance of both algorithms and the effectiveness of the two algorithms, and the results showed the best performance of the algorithms with 79.57% and 76.96% accuracy of classification of predictive models, respectively [11, 13].

In the field of healthcare data analysis, Machine learning techniques help in preference prediction. By examining different AI algorithms either alone or in conjunction with other techniques, various classification approaches can be utilized to improve the performance of classifiers [13].

## 3 Proposed model

**Figure 1:**
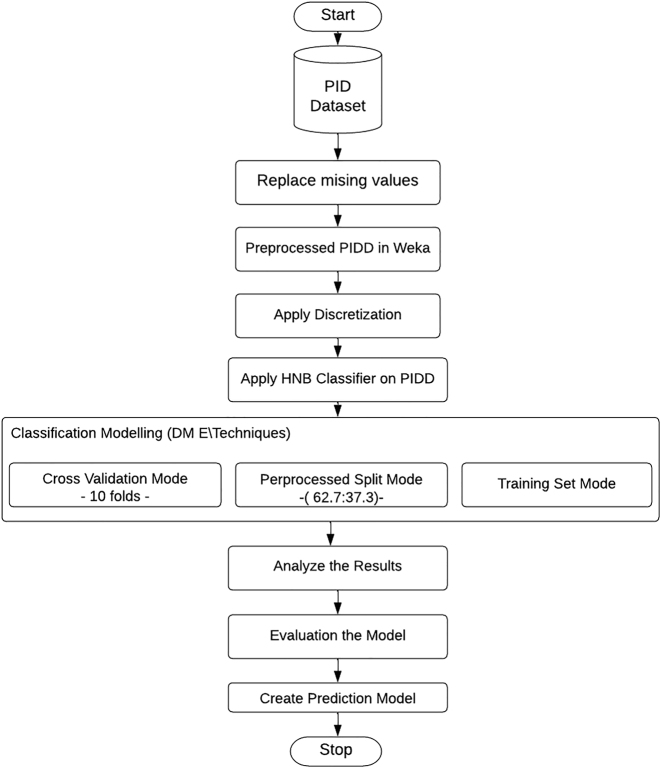
Flowchart depicting to implementation of Hidden Naïve Bayes with discretization on the PIDD for model creation.

**Figure 2:**
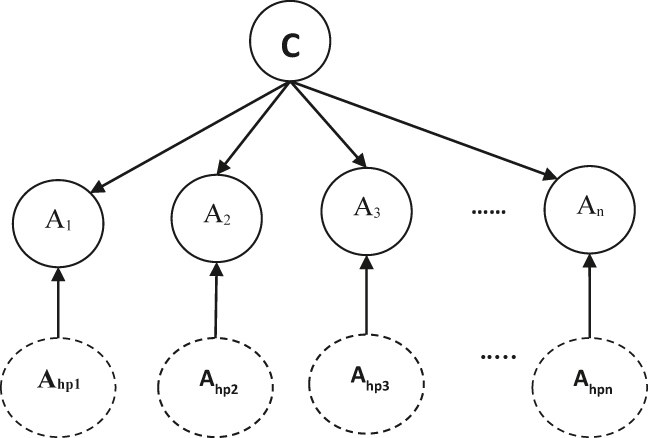
The HNB structure.

This study investigates the effectiveness of the proposed model based on the best performance of HNB by three data mining techniques (Cross-validation, Percentage Split, and Training set techniques) that demonstrate an improvement in the accuracy of diabetes prediction (Figures 1 and 2).

In the first phase, pre-processing data is required to prepare datasets used for machine learning and data mining, increasing predictive accuracy performance. The retrieved results are often out of range, missing values, and irrelevant data; at the data collection phase. A data preparation tool uses to convert dataset files into Attribute Relational File Format (ARFF). These files have data information and header, and this phase is known as the data pre-processing, which includes the under-listed steps.

### 3.1 Dataset

Diabetes dataset (Pima) is a popular database that applies the original diabetes patient datasets collected from the UCI repository, and this dataset describes females at least 21 years of age [14].

One of the Pima Indians Diabetes Datasets (PIDD) is that specific attribute values can still contribute to counts, even though their other attribute values are unknown for a training example [15]. Therefore, PIDD is typical of complex medical diagnosis issues where a small range of training and testing examples are available [5].

As shown in Table 1, there are eight numerical features in the PID dataset and the Class variable of *n* = 768 samples. The aim is to apply the eight variables to predict the Class variable. Table 2 exhibits a summary of the PID dataset considered.

**Table 1:** Features and attributes abbreviation of the Pima Indians diabetes dataset (PIDD).

Features/attributes/class	Abbreviation
Number of times pregnant	Preg
Plasma glucose concentration, 2 h in an oral glucose tolerance test	Plas
Diastolic blood pressure (mm Hg) (BP)	Pres
Triceps skinfold thickness (mm)	Skin
2-h serum insulin (mu U/mL)	Insu
Body mass index (kg/m^2^) (BMI)	Mass
Diabetes pedigree function (DPF)	Pedi
Age (years)	Age
Class variable (diabetes onset within five years)	Class

**Table 2:** Features and parameters of the Pima Indians Diabetes Dataset (PIDD).

Abbr.	Type	Mean	Standard deviation	Min	Max	Distinct	Unique
Preg	Numeric	3.8	3.4	0	17	17	2 (0%)
Plas	Numeric	120.9	32.0	0	199	136	19 (2%)
Pres	Numeric	69.1	19.4	0	122	47	8 (1%)
Skin	Numeric	20.5	16.0	0	99	51	5 (1%)
Insu	Numeric	79.8	115.2	0	846	186	93 (12%)
Mass	Numeric	23.0	7.9	0	67.1	248	76 (10%)
Pedi	Numeric	0.5	0.3	0.078	2.42	517	346 (45%)
Age	Numeric	33.2	11.8	21	81	52	5 (1%)
Class	Nominal	–	–	0	1	2	0

**Table 3:** Class parameters of the Pima Indians Diabetes Dataset (PIDD).

No	Label	Count	Weight
1	Tested negative	500.0	500.0
2	Tested positive	268.0	268.0

PIDD consist of (*N*) 500 patients of non-diabetic and 268 patients of diabetic from all cases (*n*) in PIDD (Table 3).

(1) $$f\left(x\right)=\left\{\begin{array}{c} 0\;\;\;\;\;\text{if}\;\;\;\;x=N\\ 1\;\;\;\;\;\text{if}\;\;\;\;\;x\neq N \end{array}\right.$$

where *x* is the data of the patient, *f* is a prediction of diabetes and represents (0,1) (negative, positive), that *f* is zero for all *x* = *N* and *f* is one for *x* is not equal to *N*. Therefore, to calculate the accuracy rate by mathematical.

(2) f(0,1)=N/n

Thus, the accuracy rate is 65.1%, and the error rate of 34.9% for each *x* = *N*.

### 3.2 HNB classification

Task classification takes the collection of records of each instance of a dataset and labels it as a distinct class as if it belongs to a class.

The method of HNB is focused on the concept of having a hidden parent of each attribute [16]. It inherits the structural simplicity of Naive Bayes classification (NB), which can quickly be learned without structure learning [17].

The HNB competes with state-of-the-art classification algorithms. The HNB has marked improvement over Naive Bayes and a single understandable classifier, and it is generally more efficacious [18].

*C* is the parent of all attribute nodes (Root) and the class node. Each (*A*_*i*_) attribute has a hidden parent attribute (*A*_hp*i*_), *i* = 1, 2, …, *n* for *A*_*i*_ is simply a combination of the weighted factors from all other attributes [12]. A dashed circle represents it. The arc from the hidden parent *A*_hp*i*_ to *A*_*i*_ is also represented by a dashed directed line distinguishing it from regular arcs [19].

(3) $$P\left(A_{1},\ldots ,A_{n},C\right)=P\left(C\right)\overset{n}{\underset{i=1}{\prod }}P\left(A_{i}\backslash A_{\text{hp}i},C\right)$$

(4) $$P\left(A_{i}\backslash A_{\text{hp}i},C\right)=\overset{n}{\underset{j=1\;\;j\neq i}{\sum }}Wij*P\left(A_{i}\backslash A_{\text{hp}i},C\right)$$

Where *P*(*A*_*i*_\*A*_hp*i*_, *C*) = *P*(*A*_*i*_\*A*_1_, …, *A*_*i* 1_, *A*_*i*+1_, …, *A*_*n*_, *C*) and

(5) $$\overset{n}{\underset{j=1\;\;\;j\neq 1}{\sum }}Wij*=1$$

Thus

(6) $$I_{p}\left(A_{i},A_{j}\backslash C\right)=\overset{n}{\underset{j=1\;\;\;j\neq 1}{\sum }}P\left(A_{i}\backslash A_{\text{hp}i},C\right)*\log\frac{P\left(A_{i},A_{j}\backslash C\right)}{P\left(A_{i},C\right)*P\left(A_{j},C\right)}$$

HNB classifier computes the estimated values from the PID dataset and uses *I*_*p*_(*A*_*i*_, *A*_*j*_\*C*), which calculates the information of mutual and conditional *I*_*p*_(*A*_*i*_, *A*_*j*_\*C*) among attributes *A*_*i*_ and *A*_*j*_ as the weight of *I*_*p*_(*A*_*i*_, *A*_*j*_\*C*) at a given *C*.

(7) $$W_{i}=\overset{n}{\underset{j=1\;\;j\neq 1}{\sum }}I_{p}\left(A_{i},A_{j}\backslash C\right)$$

(8) $$W_{ij}=\overset{n}{\underset{j=1\;\;\;j\neq i}{\sum }}\frac{I_{p}\left(A_{i},A_{j}\backslash C\right)}{W_{i}}$$

### 3.3 Discretization method

Discretization is a standard method in machine learning and plays a vital role in knowledge discovery and Data Mining that transforms quantitative attributes into a qualitative ones. The discretization method is divided into two sub-ranges: value range and several instances (frequency) for each interval [20].

Discretizing the real-valued attributes is essential when working with classifiers-type algorithms. It is perhaps more useful when there are natural groupings within the values of given attributes. In this proposed approach, discretization has been utalised as an instance filter in the pre-processing data stage. This is because PIDD has attribute values of continuous data, making the presented work in this study more complex, therefore, discretizing a numeric attribute range into nominal attributes in the dataset has been applied. Filters are used to remove the noisy data in data mining and applied to training and test datasets [20]. The following Table 4 shows how continuous values were divided into intervals for each attribute in PID dataset.

**Table 4:** Table of intervals for each attribute of the diabetes dataset.

Attributes	Interval (labels)	Counts and weights (instances)
Preg	−Inf ≤ 6.5	599
	6.5 ≤ +inf	169
Mass	−Inf ≤ 27.85	222
	27.85 ≤ +inf	546
Pedi	−Inf ≤ 0.527	509
	0.527 ≤ +inf	259
Age	−Inf ≤ 28.5	367
	28.5 ≤ +inf	401
Insu	−Inf ≤ 14.5	375
	14.5 ≤ 121	191
	121 ≤ +inf	202
Plas	−Inf ≤ 99.5	197
	99.5 ≤ 127.5	288
	127.5 ≤ 154.5	161
	154.5 ≤ +inf	122

There are two attributes (pals and skin) with only one interval and the other attributes are shown in Table 4 [21].

## 4 Results and discussion

No test dataset was available using Waikato Environment for Knowledge Analysis (WEKA). Hence, the percentage split is divided into X% for training data and Y% for testing data [22]. The basic assumption of machine learning is that the training and testing sets are independent and sample from an infinite population (same population). In the case of using only one dataset, it should hold part of it out for testing. So, in this proposal method based on PIDD, a slight variation in results is expected each time it is applied. Therefore, Weka produces the same results by design and making sure it reinitializes the random number generated each time.

The initial steps of the experimental work are as follows:

**Step 1**. Importing samples from the Pima Indian Data Set (diabetes dataset).

**Step 2**. Numerical data discretization.

**Step 3**. Training dataset by implementing learning algorithm techniques on the data set.

**Step 4**. Testing dataset by trained data.

**Step 5**. Carry out HNB classifier on outcomes of steps 4 and 5.

**Step 6**. Evaluating HNB classifier performance with step 3.

**Step 7**. Finally, build a predictive model.

### 4.1 Performance analysis

#### 4.1.1 Performance measures

The experiments have been evaluated using the standard matrices of Precision, F-measure, Accuracy, and Recall for the HNB classification. Define True Positive (TP) represents a correct predictions number that an instance is a Sensitivity, True Negative (TN) represents an incorrect predictions number that an instance is a Specificity, and False Negative (FN) represents the number of the incorrect predictions that an instance is a Specificity. False Positive (FP) means the number of correct predictions that an instance is a Sensitivity. Through the preceding, the predictive classification table has been calculated, as known as Matrix of Confusion Table 5 shows several negative and positive examples presented to training and testing data [23].

**Table 5:** Confusion matrix.

	Negative condition	Positive condition
Negative test results	TN	FN
Positive test results	FP	TP

Frequently-used empirical evaluation measures include accuracy, F1 score, precision, recall, specificity, and area under the curve (AUC). The ratio of the total number of predictions that were correct to the total predictions made was calculated by:

(9) Accuracy=((TN+TP)/(TN+FN+TP+FP))*100

#### 4.1.2 Performance approaches and DM techniques

There are four methods of the DM techniques used on HNB classification by discretization method of PID dataset. This study utilizes three techniques (cross-validation, percentage split, and training set) for comparison to determine the best performance and high accuracy. Testing sets from the previous three methods were excluded due to the lack of test data for the Pima diabetes dataset.

Moreover, it is rare to use the training or testing set to form the classifier. Therefore, the HNB classifier performance with the real data is evaluated in the first stage. Then, it becomes necessary before implementation to verify predictive models by detecting and understanding their characteristics and quality [24].

The classification accuracy or error rate is used to measure a classification model performance in the test set, and the classification accuracy is calculated using the above accuracy function 9. Besides, it is used to compare various classifiers’ relative performance within the same domain. Nevertheless, it is essential to know the class labels of the test data. On the other side, an evaluation method is required to measure the classification model and compute the accuracy.

**First Phase:** *Cross-validation*: is a method for evaluating a classifier model’s accuracy. Successively, it divides the data structure of the HNB classifier into subsets, creates models in subsets, and then measures the model accuracy for each subset. The cross-validation gives the model the opportunity to train on multiple train-subsets to estimate how well it will perform on unseen datasets [25]. *The 10-fold method*: is essentially different from the split method: for 10-folds, the 10 testing datasets are disjoint, which is not the case. The purpose of this is to evaluate the model performance in the absence of a large/variety of datasets, especially when there is a need to test the method on a sufficient number of real datasets. Assuming there is only one dataset, there is a need to do 10 independent experiments; therefore to design such experiments, the datasets are split into 10-folds [26]. It can be computationally expensive since each fold is used to evaluate the prediction ability of the method while using the remaining 9 folds for the model training. It is not required that 10-folds be done based on a quite random procedure to ensure that the obtained 10-folds are created quite objectively [11].

The accuracy of the models was computed from the test data set, which indicates the models offer good predictive performance, showing positive outcomes (provide numbers possibly TN and TP). Table 6 described the performance of a model of classification (HNB classifier) on the Pima dataset of the test data to use cross-validation in 10-fold mode [27].

**Table 6:** Confusion matrix of diabetes dataset using HNB classifier based on cross-validation by 10-fold mode.

Class	Tested negative	Tested positive
Tested negative	431	69
Tested positive	109	159

**Second Phase:** Training set mode is a typical technique for model evaluation, but can lack the appropriate accuracy and performance when applied to the PID datasets with an HNB classifier. It can quickly determine the HNB classifier reliability and compare models that depend on the same structure by reviewing the returned statistics [28].

**Third Phase:** Percentage Split mode; this testing method splits the dataset into sections based on the predefined percentage, whereby the algorithm applies rules learned from the training data and then applied the rules to the testing data.

A 50:50 split appears to be the most frequently used as the entire dataset is evenly split without bias. The 50:50 split (although producing slightly lower classification accuracy) has a reliable sample size and evenly distributed (splits) training and testing data [11].

A 90:10 split yields slightly better classification precision, but the sample size is too small and limited to shape a reasonable opinion based on objective evidence [25].

### 4.2 Performance evaluation

The classification was conducted on the Hidden Naïve Bayes (HNB) classifier, and accuracy was evaluated using a 10-fold cross-validation test. Cross-validation involves breaking a dataset into 10 pieces, and on each piece, testing the performance of a predictor build from the remaining 90% of the data [25]. The classification accuracy was taken as the average of the 10% predictive accuracy values.

Few experiments have been done led the percentage split (62.7%) seems to be the most reliable and accurate via distributed training and testing processes of the dataset. The results appeared to have a slightly higher classification accuracy than a percentage split of 50%. Tables 8 and 9 show the experimental results using the mode of percentage split (62.7%).

As shown in Table 9, The Negative predictive value (NPV) is 79.81% which calculates the probability that diabetes disease is not present (absent of the disease) when the result is positive. Positive Predictive Value (PPV) calculates the probability that the diabetes disease is present when the result is negative. Table 9 records accuracies obtained by three different approaches and performance measures of the HNB classifier on the PID dataset and presents the result of HNB.

Comparative results in above Tables 6 [Table j_jib-2021-0037_tab_007] [Table j_jib-2021-0037_tab_008]–9 as well as Figure 4 show that the percentage split technique side values are the highest technique (Table 10).

**Table 7: j_jib-2021-0037_tab_007:** Confusion matrix of diabetes dataset using HNB classifier based on full training mode.

Class	Tested negative	Tested positive
Tested negative	441	59
Tested positive	101	167

**Table 8: j_jib-2021-0037_tab_008:** Confusion matrix of diabetes dataset using HNB classifier based on percentage split mode.

Class	Tested negative	Tested positive
Tested negative	170	21
Tested positive	31	64

**Table 9:** Results proposed model for PID dataset accuracy, error, and other measurements of the performance of HNB.

Techniques	Cross-validation	Full training	Percentage split
Accuracy	76.80%	79.17%	81.82%
Error rate	23.18%	20.83%	18.18%
NPV	79.81%	81.37%	84.58%
FNR	40.67%	37.69%	32.63%
F-measure	83.00%	84.70%	87.00%

**Figure 3:**
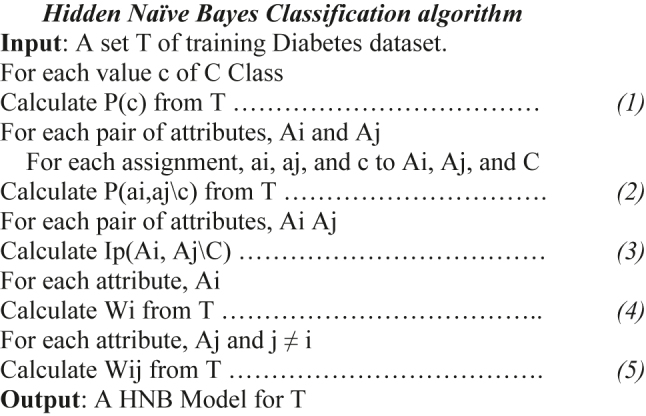
HNB algorithm for Diabetes Dataset classification.

**Figure 4:**
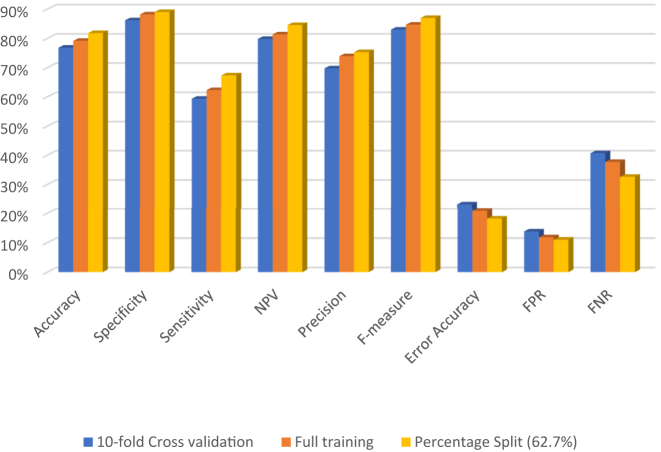
Performance measures calculation for HNB for three evaluation techniques.

**Table 10:** Prediction Accuracy of PID dataset.

Technique	Method	Accuracy
Percentage split (62.7:37.3)	HNB + discretization	82%

### 4.3 Model evaluation

#### 4.3.1 Model evaluation by indices of accuracy (accuracy evaluation)

The performance comparison of the HNB algorithm on the PID dataset was performed based on parameters such as kappa statistics, mean absolute error, relative squared error, and mean-squared error from the total of 768 testing instances. The following Table 11 provides comparative results for the above metrics. The algorithm’s performance applied to the PID dataset in Weka is given based on various factors. Kappa Statistic is a significant metric that compares Observed Accuracy with Expected Accuracy (random chance) to judge the consistency of the model. Mean Absolute Error (MAE) is the average of the absolute error between observed and forecasted values. Root Mean Squared Error (RMSE) measures the differences between values (Sample and population values) predicted by a model or an estimator and the values observed. Relative Absolute Error (RAE) represents the ratio of the absolute error of the measurement to the accepted measurement. Root Relative Squared Error (RRSE) measures [28].

**Table 11:** Performance Results of THE HNB classifier based on PIDD USING FULL training and 10-fold Cross-validation and percentage split techniques.

Techniques	Cross-validation	Full training set	Percentage split
No. instances correctly	608	608	234
No. instances incorrectly	160	160	52
Kappa statistic	0.5242	0.5242	0.5791
MAE	0.2897	0.2897	0.281
RMSE	0.3774	0.3774	0.3657
RAE	63.74%	63.74%	62.05%
RRSE	79.17%	79.17%	77.51%
Total number of instances	768	768	286

#### 4.3.2 Model evaluation by roc and AUC curve

**Figure 5:**
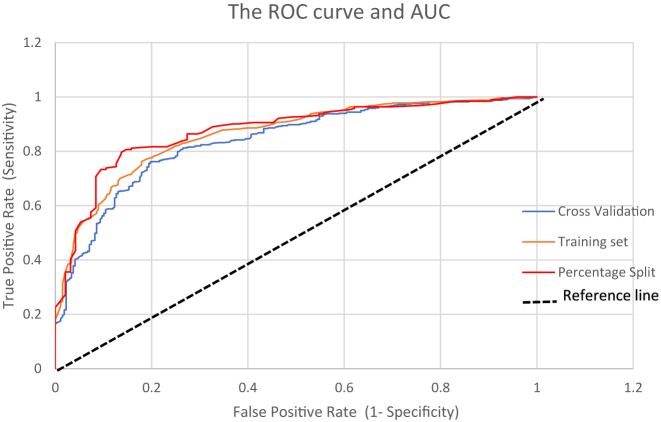
Roc curve and AUC for HNB.

ROC receiver operating characteristic curve and AUC (called Area Under the ROC curve AU-ROC) is one of the famous evaluation metrics used to assess the predictive model’s performance. The rationale for the optimal ROC curve is to get the highest true-positive rate (sensitivity) for the lowest false-positive rate (1-specificity). AUC reflects how good the test is as it distinguishes between patients with the disease and those without the disease. The results of the experiments given in Table 12, and ROC curves obtained for these tests are shown in the following Figure 5 while focusing on three evaluation techniques of data mining, the HNB classifier based on the diabetes disease detection model’s accuracy, error rate, and the AUC value.

**Table 12:** Performance evaluation based on three TECHNIQUES ON PID dataset.

Techniques	Cross validation	Full training	Percentage split
Specificity (TNR)	86.20%	88.20%	89.01%
Sensitivity (TPR)	59.33%	62.31%	67.37%
Precision (PPV)	69.74%	73.89%	75.29%
FPR	13.80%	11.80%	10.99%
Recall (TPR)	59.33%	62.31%	67.37%
ROC	0.837	0.863	0.876

Figure 5 illustrates that based on the percentage split method, the HNB classifier attempts closer to the top left corner and occupies a larger AUC than the HNB classifier based on cross-validation and training set methods. These findings indicate that the HNB classifier based on the percentage split method (shown in Red) for the diabetes detection model performs better in terms of performance measures when compared to HNB with techniques of the cross-validation (Blue) and training set methods-based model (Brown). Figure 5, shows clearly that the AUC curve for the percentage split method achieved 0.876, which is a better test for predicting diabetes than the total training set method with 0.863 and the cross-validation method with 0.837.

There are three cases in the discussion to results obtained based as shown on the plot above

**First Case** is generally recognized that the closer the AUC value to 1, the greater the accuracy of the classifier’s performance.

**The Second Case** of the reference line (baseline), as in Figure 5, indicates that the AUC equals 0.5, which is a region that cannot be distinguished from the curve due to the TPR being equal to the FPR and called random guesses.

**The Third Case** is considered the focus of the research attention and discussion.

This study represents a single point in the ROC plot. For instance, the model with parameters (FPR, TPR). Whereas when taking point on the FPR axis = 0.10, the TPR for the three evaluation techniques accentuates through different values, which represent Cross-Validation (Blue), Training set (Brown), and Percentage split (Red) on the TPR axis points, respectively (0.58, 0.63, 0.73).

Choosing a classification threshold is a crucial business decision: minimize the false positive rate and maximize the positive rate.

There is a meaningful interpretation for diabetes disease classification in certain areas under the ROC curve (AUC) determined through the bias issues and optimal cut-off values from the creation model. The ROC curve is primary in finding the optimal cut-off values; therefore, the sensitivity and specificity are computed across all possible threshold values.

The interval of (0–0.2) on the FPR axis represents the info of the prediction with higher scores, in contrast, the interval of (0.8–1) on the same axis represents the info with lower scores. Therefore, the focus is on whether the model performed well in the higher score region.

The dependency for the section of “diseased” patients obtained for the time of prediction selected over the complete dataset is represented by a curve (i.e., only those chosen for that the projected probability of having diabetes exceeds the chosen threshold).

Performance was evaluated based on various techniques on the PID dataset for HNB algorithm accuracies. The results of the HNB classifier accuracy of prediction were compared with relevant studies, which used HNB on the diabetes dataset to assess the performance criterion. Through the improvements mentioned in previous studies (see Table 13) and the tuning of performance measures. The findings show that the proposed model presented better accuracy than other methods, as shown in Table 13.

**Table 13:** Performance comparison results tested on same HNB classifier in PID dataset.

Reference	Method	Accuracy %
[29]	The author used type 2 diabetes mellitus (T2DM) with the cross-validation method in weka	74.9
[30]	Authors use adaboost to increase HNB accuracy in Weka	74.57
[31]	HNB; Weka	76
Proposed model	Percentage split and data discretization method	82

The proposed model outperforms the HNB classifier with a discretization filter and the HNB-based model for overall performance metrics. Compared to other techniques, it has greater accuracy without employing the method of feature selection, and the Sensitivity and True positive rates are higher than other comparative models [29], [30], [31].

## 5 Conclusions

The study presents the techniques of the machine learning algorithm using a proposed classification model (HNB). The HNB classifier tested one of the intensively researched problems in healthcare which is the diagnosis of Pima diabetes. The model applies the discretization method and the transformation of real value data into nominal value data for the diabetes dataset (use of dataset as interval), which was implemented in the Weka framework. The HNB classifier performance of the three techniques was investigated for diabetes disease prediction, demonstrating the proposed model’s efficacy for prediction. The findings demonstrate that the model reaches the best performance based on the percentage split technique for diabetes disease. A classifier model based on the HNB algorithm with a discretization method has been introduced to reduce the NB naivety assumption. The results obtained indicate that a less complex model with an AUC-ROC curve of 0.86 can be achieved with Percentage Split compared to other techniques, namely Cross-validation and Training Set. Experiment results show that the diabetes disease prediction model was improved by the discretization method using the percentage split technique by 62.7%, achieving an accuracy of 82% in diabetes disease prediction when compared with other models as shown in Table 13. This study concludes that the predictive model can contribute to future experiments, considering that the proposed model is best suited as a decision support system for diabetes disease diagnosis.

Since diabetes is a complex disease. Many factors such as race, gender, age, and environment may correlate with the risk of diabetes. An extension of this work in the near future will focus on developing HNB classifier performance using feature selection techniques instead of the discretization method, in order to increase the detection rate of the final classification of diabetes. Additionally, it may focus more on automatically estimating the disease severity that has been detected.
